# Functional and Clinical Impact of CircRNAs in Oral Cancer

**DOI:** 10.3390/cancers12041041

**Published:** 2020-04-23

**Authors:** Ion Cristóbal, Cristina Caramés, Jaime Rubio, Marta Sanz-Alvarez, Melani Luque, Juan Madoz-Gúrpide, Federico Rojo, Jesús García-Foncillas

**Affiliations:** 1 Cancer Unit for Research on Novel Therapeutic Targets, Oncohealth Institute, IIS-Fundación Jiménez Díaz-UAM, E-28040 Madrid, Spain; ccarames@fjd.es (C.C.); jaime.rubiop@quironsalud.es (J.R.); 2 Translational Oncology Division, Oncohealth Institute, IIS- Fundación Jiménez Díaz-UAM, E-28040 Madrid, Spain; 3 Medical Oncology Department, University Hospital “Fundación Jiménez Díaz”, UAM, E-28040 Madrid, Spain; 4 Pathology Department, IIS- Fundación Jiménez Díaz-UAM, E-28040 Madrid, Spain; marta.sanza@quironsalud.es (M.S.-A.); melani.luque@quironsalud.es (M.L.); jmadoz@fjd.es (J.M.-G.); frojo@fjd.es (F.R.)

**Keywords:** circRNA, signaling, diagnosis, prognosis, oral cancer

## Abstract

The increasing number of recently published works regarding the role of circular RNAs (circRNAs) in oral cancer highlights the key contribution of this novel class of endogenous noncoding RNAs as regulators of critical signaling pathways and their clinical value as novel biomarkers. This review summarizes and puts into context the existing literature in order to clarify the relevance of circRNAs as novel mediators of oral cancer pathogenesis as well as their potential usefulness as predictors of clinical outcome and response to therapy in this disease.

## 1. Introduction

**Figure 1:**
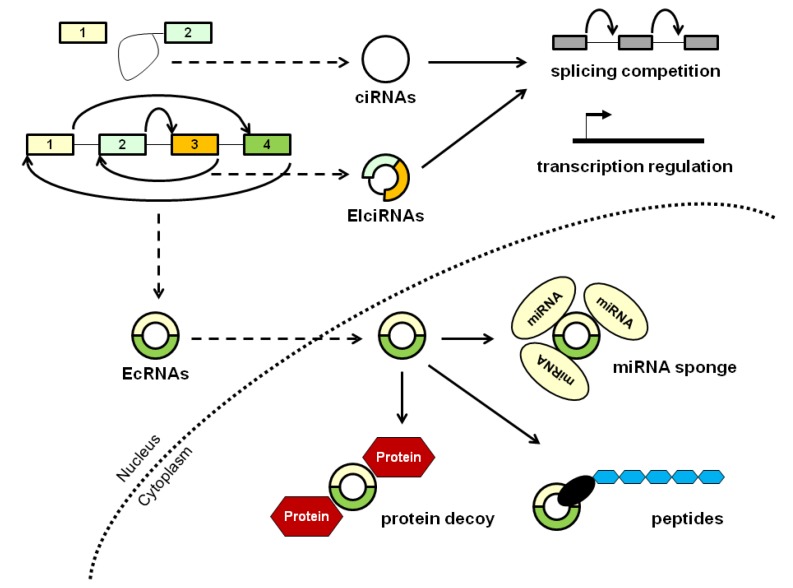
Biogenesis and molecular functions of circular RNAs (circRNAs).

Oral cancer represents the eighth most common cancer and a major health problem worldwide with an estimated annual incidence of more than 500,000 cases [1,2]. Oral squamous cell carcinoma (OSCC) accounts for more than 90% of tumors in the oral cavity. Although there have been advances in screening and treatment of oral cancer in the last decades, patients are often diagnosed at late stages, which compromises the outcome. In fact, it is a very aggressive disease with high rates of recurrence and metastasis and a five-year survival rate of only around 20% at late stages (III–IV) compared to 80% in early stages (I–II) [3,4,5]. The main risk factors are alcohol consumption and smoking, together with other causes such as diet, viral infections, and poor oral hygiene [6]. Despite many years of investigation and significant advances in therapeutic strategies, especially in surgical methods and chemoradiotherapy, the molecular mechanisms underlying OSCC pathogenesis remain unclear, and there is a lack of efficient established markers for early diagnosis and prognosis of this disease. Circular RNAs (circRNAs) are a novel class of noncoding RNAs characterized by a covalently closed loop without a 5′ cap or a 3′ poly(A) tail. They are generated from exonic and/or intronic sequences of primary transcripts by back-splicing, in which the spliceosome links a 5′ donor splice site to an upstream 3′ acceptor splice site [7]. There are three major classes of circRNAs depending on their origin: exonic circRNAs (EcRNAs), when all introns are removed; exon–intron circRNAs (EIcRNAs), when some intronic sequences are retained; and circular intronic RNAs (ciRNAs) [8]. Although most circRNAs are predominantly located in the cytoplasm, ciRNAs and EIcRNAs play important roles in the nucleus, such as transcription regulation or competition with linear RNA splicing. In the cytoplasm, circRNAs can function as post-transcriptional regulators, acting as microRNA (miRNA) sponges and thereby impairing miRNA-mediated gene suppression, or serve as protein decoys binding and sequestering proteins. Moreover, some circRNAs have an open reading frame and translate regulatory peptides [9]. CircRNA biogenesis and functions are illustrated in Figure 1.

In fact, their closed structure makes circRNAs more stable and resistant to degradation as they are not affected by RNAse exoenzymes [10]. CircRNAs were identified for the first time in Sendai viruses in 1976 and were observed in eukaryotic cell cytoplasm by electron microscopy a few years later. They were initially considered as a product of RNA mis-splicing [11,12,13,14]. They are conserved and widely expressed among mammals and play key roles in many biological and pathological processes, including human cancer [10,15,16]. At the molecular level, circRNAs are enriched in miRNA binding sites, thereby acting as miRNA sponges and impairing the inhibitory effects of specific miRNAs on their target mRNAs. Moreover, they can also function as transcriptional regulators of competing endogenous RNAs [17,18,19,20]. An increasing number of studies have shown that expression of circRNAs is altered in human cancer. They can function as oncogenes or tumor suppressors downstream depending on their targets [21]. In their upstream biogenesis, it has been demonstrated that splicing factors bind the flanking intron–exon region, facilitating the back-splicing of circRNAs [22,23].

## 2. CircRNAs Acting as Oncogenes

### 2.1. Circ_0002185

**Figure 2:**
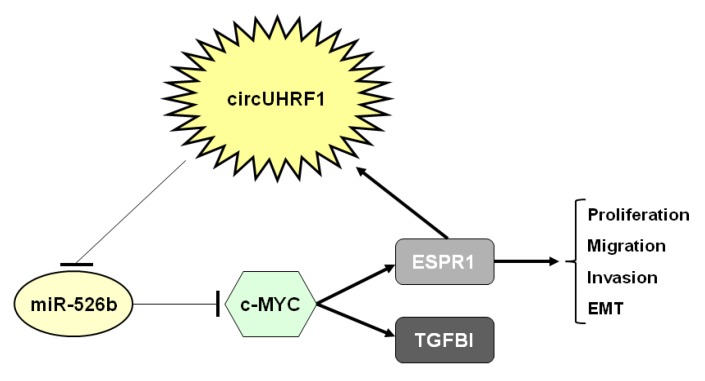
Molecular mechanism of circUHRF1.

This circRNA, also called circUHRF1, was found to be upregulated in OSCC tissues and showed oncogenic roles in this disease. Stable silencing of circ_0002185 in SCC25 and CAL27 cells using specific shRNAs resulted in inhibited proliferation, migration, and invasion abilities. Knockdown of this circRNA also led to repressed epithelial-to-mesenchymal transition (EMT), decreasing the expression of N-cadherin and vimentin and enhancing E-cadherin levels. These results were confirmed by the ectopic expression of circ_0002185 in both cell lines. In addition, circ_0002185 knockdown inhibited tumor volume and weight in an in vivo xenograft model using SCC25 cells. Mechanistically, circUHRF1 acted as a miR-526b-5p sponge, thereby positively modulating the transcription factor c-MYC, which induces the expression of both *TGF-β1* and *ESRP1*. The splicing factor ESRP1 targeted the flanking introns and promoted the circularization and biogenesis of circ_0002185, leading to the formation of a circUHRF1/miR-526b-5p/c-Myc/TGF-β1/ESRP1 feedback loop (Figure 2) [24].

### 2.2. Circ_0001821

Circ_0001821, alternatively named circPVT1, is derived from the exon 3 of the oncogene *PVT1*, which is located on chr8:128902834–128903244, a cancer susceptibility locus [25,26]. It has been shown to play oncogenic roles in several tumor types [27]. In OSCC, this circRNA has been found to be overexpressed in both OSCC cell lines and tumor tissues compared to normal keratinocytes and tissues, respectively. He et al. [28] demonstrated that circPVT1 includes two specific binding sites for miR-125b, leading to the overexpression of the miR-125b downstream target signal transducer and activator of transcription 3 (*STAT3*) through the formation of a circPVT1/miR-125-b/*STAT3* signaling axis. Thus, circPVT1 functions as a competing endogenous RNA to increase *STAT3* levels and proliferation by sponging miR-125b.

### 2.3. Circ_100290

Circ_100290 has been reported to function as an oncogene in some cancers. Thus, this circRNA has been described to promote colorectal cancer progression, functioning as a competing RNA of *FZD4* by sponging miR-516, which leads to the activation of the Wnt/β-catenin pathway [29]. Moreover, circ_100290 has been found to enhance proliferation and impair apoptosis in acute myeloid leukemia via sponging miR-203 [30]. Recently, it has been reported that this circRNA induces laryngeal squamous cell carcinoma progression by targeting the miR-136-5p/RAP2C signaling axis [31]. In concordance with these observations, Chen and colleagues showed that both circ-100290 and GLUT1 were overexpressed in 20 OSCC tumor samples and in the Tca8113 and CAL27 cell lines in comparison with noncancerous tissue specimens and normal keratinocytes. Circ_100290 silencing enhanced apoptosis, inhibited colony formation ability of OSCC cells, and reduced lactate production, while it increased the glucose in the culture medium due to GLUT1 downregulation. Next, the authors demonstrated that circ_100290 targets miR-378a, which directly targets *GLUT1*, highlighting the existence of a circ_100290/miR-378a/*GLUT1* axis that regulates both cell growth and glycolisis in OSCC cells [32].

### 2.4. Circ_0001742

Two recently published works have explored the role of circ_0001742 in tongue squamous cell carcinoma (TSCC). Shao et al. showed that circ_0001742 was overexpressed in 23 TSCC patients and correlated with advanced clinical stage and positive lymph node metastasis. Ectopic downregulation of this circRNA decreased proliferation, invasion, and epithelial-to-mesenchymal transition in both TCA8113 and SCC9 cells. The molecular mechanism involved the sponging of miR-634 by this circRNA and the formation of a circ_0001742/miR-634/*RAB1A* signaling axis that was targeted by circ_0001742, thereby promoting TSCC progression [33]. Furthermore, Hu and colleagues confirmed the overexpression of circ_0001742 in TSCC patients and also observed that knockdown of this circRNA resulted in inhibited proliferation, migration, and invasion as well as increased apoptosis of TSCC cells. However, they identified the miR-431-5p as a direct target of circ_0001742 and the formation of circ_0001742/miR-431-5p/*ATF3*, in which the overexpression of ATF3 rescued the effects mediated by miR-431-5p [34]. Therefore, it remains necessary to further clarify the contribution of each signaling axis in TSCC progression.

### 2.5. Circ_0059655

In order to evaluate the significance of circRNAs in the pathogenesis of salivary adenoid cystic carcinoma (SACC), 10 tumor tissues and their paired normal submandibular gland tissues were included to perform ceRNA microarrays, including mRNAs, lncRNAs, and circRNAs, as well as miRNA arrays. The results of the arrays included 3792 mRNAs, 7649 lncRNAs, 11,553 circRNAs, and 132 differentially expressed miRNAs. The ceRNA regulatory network analysis showed a potential relationship between circ_0059655 and miR-338-3p. The authors experimentally demonstrated that circ_0059655 acts as a sponge of miR-338-3p to form a circ_0059655/miR-338-3p/*CCND1* axis. Functionally, knockdown of circ_0059655 led to reduced proliferation, migration, and invasion abilities of SACC83 cells, highlighting the role of this circRNA as an oncogene in SACC [35].

### 2.6. CircHIPK3

CircHIPK3 has been reported to play a role as an oncogene in numerous cancer types [36]. In concordance, the expression of circHIPK3 was found to be upregulated in OSCC tissues compared to adjacent noncancerous tissues and correlated with advanced TNM stage and higher tumor grade. The levels of circHIPK3 in SCC15 and CAL27 cells was higher than in normal keratinocytes, and circHIPK3 knockdown led to reduced cell proliferation in both cell lines. Moreover, the authors claimed that circHIPK3 acts as a sponge of miR-124 and showed that miR-124 downregulation is able to rescue the phenotype induced after circHIPK3 knockdown in OSCC cells [37].

### 2.7. Circ_0001971

The work by Tan et al. showed that circ_0001971 is upregulated in both OSCC cell lines and patient samples, which is in concordance with previous observations, indicating that this circRNA may serve as a novel salivary biomarker with diagnostic value in OSCC [38]. Of relevance, circ_0001971 knockdown decreased cell proliferation, migration, and invasion, enhanced both apoptosis and sensitivity of OSCC cells to cisplatin, and impaired in vivo tumor growth in a xenograft model. Mechanistically, circ_0001971 was found to sponge miR-194 and miR-204, and inhibition of these miRNAs reversed circ_0001971-mediated effects [39]. 

### 2.8. CircDOCK1

The involvement of circDOCK1 in OSCC apoptosis was identified in an apoptotic model of OSCC using TNF-α, in which the expression of circRNAs was compared between apoptotic and nontreated cells. Validation experiments showed that knockdown of circDOCK1 led to increased apoptosis of OSCC cells. Bioinformatics predicted interactions between circRNAs, miRNAs, and mRNAs, and a potential circDOCK1/miR-196-5p/*BIRC3* axis was proposed. Both circDOCK1 silencing or miR-196-5p overexpression led to increased apoptosis and decreased BIRC3 expression levels as this circRNA acts as a miR-196-5p sponge, thereby regulating *BIRC3* and modulating apoptosis of OSCC cells [40]. These results showing the role of circDOCK1 as an oncogene in OSCC are in concordance with previous results indicating that circDOCK1 promotes bladder cancer progression [41].

A list of the different reported circRNAs with oncogenic or tumor suppressor roles in oral cancer and the pathways affected are summarized in Table 1.

A summary of the different circRNAs involved in the regulation of each cellular process is given in Table 2.

## 3. CircRNAs Acting as Tumor Suppressors

### 3.1. Circ_0002203

Significant low expression of circ_0002203 was observed in tumor tissues from a cohort of 40 OSCC patients compared to their corresponding adjacent tissues, and the expression in OSCC cell lines was lower than in human oral keratinocytes. Ectopic expression of circ_0002203 after lentiviral transfection of SCC15 and CAL27 impaired proliferation, migration, and invasion abilities in vitro and reduced tumor growth in an in vivo model using SCC15 cells [42]. However, the molecular mechanisms involved in these circ_0002203-mediated effects remain to be investigated. 

### 3.2. Circ_0004491

In a recent work, circ_0004491 was found to be significantly downregulated in a series of 40 OSCC tumor tissues compared with their paired normal tissues, and its downregulation correlated with lymph node metastasis. Functionally, circ_000449 markedly affected invasion and migration abilities of OSCC cells as well as epithelial- to-mesenchymal transition as it was associated with increased E-cadherin and decreased vimentin expression [43]. Circ_0004491 may therefore play a role in the progression of OSCC as a tumor suppressor.

### 3.3. CircFLNA

Filamin A (FLNA)-derived circRNA (circFLNA) was found to be downregulated in tumor tissues in five OSCC patients compared with their corresponding normal tissues. Moreover, Zhang and colleagues described in their work that circFLNA expression was induced after treatment with fucoidan, a sulfated polysaccharide with reported antitumor properties, in both SCC15 and SCC25 cells and led to decreased cell proliferation, migration, and invasion abilities and enhanced cell apoptosis in SCC15 cells [50]. These findings suggest the tumor suppressor role of circFLNA in OSCC, which is in contrast to a recent work describing that circFLNA overexpression contributes to laryngeal squamous cell carcinoma migration by sponging miR-486-3p [44].

### 3.4. Circ_0063772

Wang et al. recently reported lower circ_0063772 levels in OSCC tumor tissues than in adjacent normal controls as well as in the SCC15, SCC25, SCC9, and CAL27 cell lines than in normal keratinocytes. Moreover, the overexpression of this circRNA using lentiviral transfection impaired cell proliferation, migration, and invasion abilities of SCC15 and CAL27 cells. Of relevance, circ_0063772 overexpression led to reduced tumor volume and weight in nude mice, suggesting that this circRNA functions as a tumor suppressor in OSCC [45].

### 3.5. Circ_0070401

It has recently been reported that circ_0070401, also known as circPDK2, plays a tumor suppressor role in OSCC. Firstly, the authors analyzed circRNA expression profiles by performing microarrays in four paired OSCC samples, observing a total of 107 abnormally expressed circRNAs (28 upregulated and 79 downregulated) in tumor tissues compared to the normal tissues. They focused their study on evaluating circPDK2 in OSCC progression and validated the observed circPDK2 downregulation in a series of 56 OSCC patients. Moreover, this alteration correlated with lower differentiation grade, advanced stage, positive lymph node metastasis. Functionally, circPDK2 reduced cell proliferation of SCC15 cells in a CCK-8 assay as well as migration and invasion in the corresponding transwell assays. Flow cytometry analysis showed that this circRNA also induced apoptosis and cell cycle arrest on the G1/S phase. Validation of these observations was carried out by circPDK2 silencing, which exhibited the opposite responses. To investigate whether the overexpression of this circRNA affects tumor growth in vivo, they performed a xenograft model subcutaneously injected with SCC15 cells ectopically expressing circPDK2 and observed a marked inhibition of tumor growth. Finally, circPDK2 was identified as a sponge of miR-204-3p and increased the expression of adenomatous polyposis coli 2 (*APC2*), which is a direct miR-204-3p target, thereby impairing the oncogenic effects of miR-204-3p-mediated *APC2* inhibition through a circPDK2/miR-204-3p/*APC2* signaling axis [46].

### 3.6. Circ_0005379

Researchers conducted a comprehensive analysis of circRNAs in OSCC using high-throughput transcriptome sequencing and identified significantly lower expression of circ_0005379 in 37 OSCC tumor tissues than in paired normal controls and correlation with larger tumor size and poor differentiation. Circ_0005379 overexpression decreased proliferation, promoted apoptosis, and inhibited cell migration and invasion abilities. Moreover circ_0005379 affected epithelial-to-mesenchymal transition, decreasing the expression of N-cadherin and vimentin and increasing E-cadherin in OSCC25 and CAL27 in vitro, and also led to a reduced tumor growth in vivo. Interestingly, the authors showed that circ_0005379 enhanced sensitivity of OSCC cells to cetuximab, probably due to its involvement in the regulation of the epidermal growth factor receptor (EGFR) pathway [47].

### 3.7. Circ_0007059

The study of the circRNA expression profiles was performed in eight OSCC patients and eight normal controls using high-throughput sequencing, and circ_0007059 downregulation was detected in OSCC samples. These results were confirmed by real-time PCR in a cohort of 52 OSCC cases, and circ_0007059 was found to correlate with positive lymph node metastasis. Moreover, ectopic expression of this circRNA reduced proliferation, migration, and invasion abilities and promoted apoptosis in both SCC15 and CAL27 cells [48], which is in concordance with the tumor suppressor role described for this circRNA in lung cancer [51]. To further validate their findings, Su et al. performed a xenograft model, observing that circ_0007059 reduced tumor growth in vivo. Finally, this circRNA was proposed to act through the regulation of AKT/mTOR signaling [48]. However, its direct miRNA target to mediate these effects still remains to be determined. 

### 3.8. Circ_0012342

Lu and colleagues used microarrays to analyze the expression profiles of mRNAs, lncRNAs, and circRNAs in four samples of mucoepidermoid carcinoma of salivary gland (MECSG) and their matched nontumoral tissues, detecting a total of 3612 mRNA, 3091 lncRNAs, and 284 circRNAs with altered expression. Regarding circRNAs, the top five candidates differentially expressed in microarrays were validated by real-time PCR, and circ_0012342 downregulation was the most consistent expression pattern with the microarray results. They showed the highest fold change in MECSG compared to normal controls, suggesting its potential tumor suppressor role in MECSG [49]. Bioinformatics analysis showed let-7e-5p, miR-107, and miR-214-3p as potential targets of circ_0012342, but these candidates need to be experimentally confirmed in future works.

## 4. CircRNAs with Clinical Impact as Novel Biomarkers in Oral Cancer

A summary of the circRNAs that have been reported as novel biomarkers with clinical significance in oral cancer is given in Table 3. Of note, all of them have been reported in OSCC.

### 4.1. Circ_0086414

The expression levels of circ_0086414 were measured by real-time PCR in 55 OSCC cases and OSCC cells and were found to be significantly downregulated in tumor tissues compared with adjacent healthy tissues and normal cells, respectively. Moreover, low circ_0086414 levels were significantly correlated with higher TNM stage, larger tumor size, and positive lymph node metastasis. The area under the receiver operating characteristic curve (ROC) was 0.749, and this circRNA was proposed as a potential diagnostic biomarker in OSCC [52]. In concordance with these results, a two-circRNA signature consisting of circ_0086414 and circ_0005962 has been described to serve as a diagnostic biomarker in lung adenocarcinoma [58]. Furthermore, Li and coworkers performed functional enrichment and integrated analyses as well as the construction of a circ_0086414-miRNAs-mRNAs network using the Gene Ontology, Disease Oncology, and Kyoto Encyclopedia of Genes and Genomes, identifying 168 miRNAs and mRNAs related to circ_0086414. In fact, their results showed that circ_0086414 might be correlated with AMPK and cAMP signaling in OSCC patients by influencing the expression of mTOR, SIRT1, AKT, CaMKK2, and RHEB [52]. These observations suggest that this circRNA could act as a tumor suppressor in OSCC, but its biological effects remain to be experimentally determined and its potential diagnostic value further confirmed in forthcoming studies.

### 4.2. Circ_0002185

This circRNA, also called circUHRF1, was derived from the *UHRF1* gene (chr19:4950622–4951008) and generated via back-splicing of exons 12–13. Zhao and colleagues recently reported that this circRNA was upregulated in OSCC patients compared to normal controls. Moreover, the subgroup of OSCC patients with high circ_0002185 expression levels showed significantly shorter survival rates than cases with low levels of this circRNA [24].

### 4.3. Circ_0001821

This circRNA was found to be upregulated in both CAL27 and SCC9 cells compared to normal human keratinocytes as well as in tumor tissues in a cohort of 50 OSCC patients compared to paired normal controls. Moreover, high circ_0001821 levels correlated with larger tumor size and positive lymph node and metastasis. Finally, the authors evaluated the potential diagnostic value of circ_0001821 by establishing a ROC curve and observed that the area under the curve was 0.787, while the sensitivity and specificity were 68.6 and 86%, respectively [28]. Of note, the diagnostic value of this circRNA has previously been proposed in lung and gastric cancers [59,60].

### 4.4. Circ_0092125

The expression of circ_0092125 was significantly downregulated in 86 OSCC tissues compared with their normal adjacent matched tissues as well as in the OSCC cell lines SCC15, SCC25, and CAL27 compared to the human oral keratinocyte cell line HOK. Low circ_0092125 expression was associated with larger tumor size, higher TNM stage, and positive lymph node metastasis. Kaplan–Meier survival analyses showed that OSCC patients with low circ_0092125 expression had a significantly shorter overall survival. Moreover, Cox analyses identified circ_0092125 expression, tumor size, TNM stage, and lymph node metastasis as independent risk factors for OSCC outcome [53]. Altogether, these results indicate that circ_0092125 downregulation could serve as a marker of poor prognosis in OSCC patients.

### 4.5. CircMAN1A2

Chu-Mei and colleagues evaluated the potential clinical significance of circMAN1A2 as serum biomarker in several tumor types, including oral cancer. In fact, a cohort of 55 oral cancer patients was analyzed, and upregulation of circMAN1A2 was observed. The area under the ROC curve for this circRNA was used to evaluate its clinical diagnostic value and showed a value of 0.779. Therefore, circMAN1A2 could serve as an effective novel diagnostic serum biomarker in liquid biopsies from oral cancer patients [54].

### 4.6. Circ_0072387

The expression levels of circ_0072387 was analyzed by real-time PCR in a cohort of 63 paired OSCC tissues, three OSCC cell lines (SCC15, SCC25, and CAL27), and normal keratinocytes. The researchers observed that circ_0072387 was significantly downregulated in OSCC tumor tissues and cell lines and correlated with advanced TNM stage. The area under the ROC curve was 0.746, suggesting the potential diagnostic value of circ_0072387 in OSCC. Bioinformatics analyses predicted miR-29-3p, miR-129-3p, and miR-141-3p as potential targets of this circRNA, but experimental confirmation in future studies is required [55].

### 4.7. Circ_0008309

The authors performed high-throughput sequencing in eight OSCC cases with paired tumor and normal tissue, detecting 16 circRNAs with altered expression. The detected downregulation of circ_0008309 was validated by real-time RT-PCR in a cohort of 45 OSCC cases and correlated with poor pathological differentiation. The diagnostic value of this circRNA was analyzed via the ROC curve and showed an area under the curve of 0.764, indicating that circ_0008309 is closely associated with OSCC. Moreover, ectopic expression of circ_0008309 was found to increase ATXN1 expression in OSCC cells as well as reduce the expression of miR-136-5p and miR-382-5p. However, the role of this circRNA in sponging these miRNAs and forming a circ_0008309/miR-136-5p/miR-382-5p/*ATX1* pathway remains to be experimentally demonstrated [56].

### 4.8. Circ_001242

The expression levels of circ_001242 were measured by real-time RT-PCR in a set of 40 OSCC cases and four OSCC cell lines (SCC9, SCC15, SCC25, and CAL27). Circ_001242 was observed to be significantly downregulated in OSCC tumors compared to normal controls and in OSCC cell lines in comparison with normal keratinocytes. Moreover, circ_001242 expression negatively correlated with both tumor size and stage. A ROC curve was used to analyze the diagnostic value of circ_001242. The researchers observed an area under the curve of 0.784, indicating that this circRNA may serve as a potential novel biomarker for the diagnosis of OSCC [57].

### 4.9. Circ_0001874/circ_0001971

Zhao and colleagues performed a microarray analysis in three OSCC patients and three healthy donors to identify differentially expressed circRNAs in saliva. They observed 12 circRNAs upregulated and 20 downregulated in the saliva from OSCC cases, which were further validated by real-time RT-OCR. The authors reported that salivary circ_0001874 was upregulated and correlated with tumor stage and grade and that circ_0001971 was upregulated and associated with TNM stage. Interestingly, the combination of both circRNAs showed an area under the ROC curve of 0.922, and both circ_0001874 and circ_0001971 expression decreased in postoperative compared to preoperative samples. Altogether, these results highlight the high potential usefulness of circ_0001874 and circ_0001971 as novel salivary biomarkers for OSCC diagnosis [38].

## 5. Conclusions

Numerous circRNAs have been reported as key regulators of critical pathways in many human cancers. Although further investigation is required to clarify the role that this novel class of noncoding RNAs plays in the tumor cell, an increasing number of studies in previous years have highlighted their functional and clinical relevance in oral cancer progression. However, there are currently two limitations to consider circRNAs as clinical biomarkers in oral cancer. First, all of the studies published to date involving circRNA in oral cancer have been performed with a relatively small number of patient samples. Second, there is a lack of confirmatory studies that reproduce and validate the works describing circRNAs with a functional or clinical impact in oral cancer. Therefore, there are some circRNAs that may serve as novel, useful biomarkers with diagnostic and prognostic value, but further studies using independent and large patient cohorts are required to verify the reproducibility of the results obtained so far and confirm the functional role of circRNAs in this disease.

Although circRNAs have been progressively emerging as promising diagnostic and prognostic biomarkers in several human cancers, especially as noninvasive markers for liquid biopsies, many critical considerations have to be addressed before considering their inclusion in the clinical routine. Thus, multicenter prospective and retrospective studies focused on screening more circRNA biomarker candidates and validating them in large patient cohorts are required to properly evaluate their clinical usefulness. Moreover, another essential issue concerns the development of reliable methods with high specificity and sensitivity to detect circRNAs in patient samples. Finally, confirmation of circRNAs as novel molecular targets in preclinical models using patient-derived xenografts would clarify their potential therapeutic value in order to develop alternative strategies based on these molecules.

## Figures and Tables

**Table 1:** Signaling pathways regulated by circRNAs in oral cancer.

CircRNA	Function	Tumor	Pathway	References
Circ_0002185	Oncogene	OSCC ^1^	circUHRF1/miR-526-5p/c-Myc/*TGFB1*/*ESRP1* loop	[24]
Circ_0001821	Oncogene	OSCC	circPVT1/miR-125b/*STAT3*	[28]
Circ_100290	Oncogene	OSCC	circ_100290/miR-378a/*GLUT1*	[32]
Circ_0001742	Oncogene	TSCC ^2^	circ_0001742/miR-431-5p/*ATF3*	[34]
Circ_0059655	Oncogene	SACC ^3^	circ_0059655/miR-338-3p/*CCND1*	[35]
CircHIPK3	Oncogene	OSCC	circHIPK3/miR-124	[37]
Circ_0001971	Oncogene	OSCC	circ_0001971/miR-194/miR-204	[39]
CircDOCK1	Oncogene	OSCC	circDOCK1/miR-196-5p/*BIRC3*	[41]
Circ_0002203	TS ^4^	OSCC	Unknown	[42]
Circ_0004491	TS	OSCC	Unknown	[43]
CircFLNA	TS	OSCC	circFLNA/miR-486-3p	[44]
Circ_0063772	TS	OSCC	Unknown	[45]
Circ_0070401	TS	OSCC	circPDK2/miR-204-3p/*APC2*	[46]
Circ_0005379	TS	OSCC	EGFR pathway	[47]
Circ_0007059	TS	OSCC	AKT/mTOR pathway	[48]
Circ_0012342	TS	MECSG ^5^	Unknown	[49]

^1^ OSCC: oral squamous cell carcinoma; ^2^ TSCC: tongue squamous cell carcinoma; ^3^ SACC: salivary adenoid cystic carcinoma; ^4^ TS: tumor suppressor; ^5^ MECSG: mucoepidermoid carcinoma of the salivary gland.

**Table 2:** CircRNAs as regulators of key cellular properties in oral cancer.

Functional Role	CircRNAs
Proliferation	circUHRF1, circPVT1, circ_100290, circ_0001742, circ_0059655, circHIPK3, circ_0001971, circ_0002203, circFLNA, circ_0063772, circ_0070401, circ_0005379, circ_0007059
Migration	circUHRF1, circ_0059655, circ_0001971, circ_0002203, circ_0004491, circFLNA, circ_0063772, circ_0070401, circ_0005379, circ_0007059
Invasion	circUHRF1, circ_0001742, circ_0059655, circ_0001971, circ_0002203, circ_0004491, circFLNA, circ_0063772, circ_0070401, circ_0005379, circ_0007059
Apoptosis	circ_100290, circ_0001742, circ_0001971, circDOCK1, circFLNA, circ_0070401, circ_0005379, circ_0007059
EMT ^1^	circUHRF1, circ_0001742, circ_0004491, circ_0005379
Tumor growth in vivo	circUHRF1, circ_0001971, circ_0002203, circ_0063772, circ_0070401, circ_0005379, circ_0007059
Drug resistance	circ_0001971 (cisplatin), circ_0005379 (cetuximab)

^1^ EMT: epithelial-to-mesenchymal transition.

**Table 3:** CircRNAs reported as novel biomarkers in oral squamous cell carcinoma (OSCC).

CircRNA	Expression	Sample	Clinical Value	References
Circ_0086414	Low	Tumor	Diagnostic	[52]
Circ_0002185	High	Tumor	Prognostic	[24]
Circ_0001821	High	Tumor	Diagnostic	[28]
Circ_0092125	Low	Tumor	Prognostic	[53]
CircMAN1A2	High	Serum	Diagnostic	[54]
Circ_0072387	Low	Tumor	Diagnostic	[55]
Circ_0008309	Low	Tumor	Diagnostic	[56]
Circ_001242	Low	Tumor	Diagnostic	[57]
Circ_0001874/circ_0001971	High	Saliva	Diagnostic	[38]
